# Structural Determinants in the Calcitonin Receptor-Like Receptor (Crlr) Important for Cgrp and Adrenomedullin (Am) Receptor Function of Crlr/Receptor-Activity-Modifying Protein (Ramp) 1 and Crlr/Ramp2 Heterodimers

**DOI:** 10.1100/tsw.2001.405

**Published:** 2001-11-15

**Authors:** W. Born, K. Leuthauser, R. Gujer, R. Muff, J.A. Fischer

**Affiliations:** Research Laboratory for Calcium Metabolism, University of Zurich, Klinik Balgrist, 8008 Zurich, Switzerland

## Abstract

Cell surface protein cross-linking, coimmmunoprecipitation, and confocal microscopy identified CRLR/RAMP1-, CRLR/RAMP2-, and calcitonin receptor isotype 2 (CTR2)/RAMP1 heterodimers as CGRP-, AM-, and CGRP/amylin receptors, linked to cAMP production. Along these lines, effects of structural alterations in the N-terminal extracellular domain of the human CRLR on cell surface expression as well as the association with RAMP and CGRP or AM have been investigated.

